# Supplementary material for: Characterizing the deep uncertainties surrounding coastal flood hazard projections: A case study for Norfolk, VA
Source: Sci Rep. 2019 Aug 6;9:11373. doi: 10.1038/s41598-019-47587-6 (PMC6684642; doi:10.1038/s41598-019-47587-6)
Supplement: Supplementary file 1 — Supporting Information [file 41598_2019_47587_MOESM1_ESM.pdf]

Supporting Information for: Characterizing the deep uncertainties surrounding coastal flood hazard projections: A case study for Norfolk, VA

Kelsey L. Ruckert[1], Vivek Srikrishnan[1], and Klaus Keller[1,2,*]

[1]Earth and Environmental Systems Institute, The Pennsylvania State University, University Park, PA, 16802, USA

[2]Department of Geosciences, The Pennsylvania State University, University Park, PA, 16802, USA

[*]Corresponding author: klaus@psu.edu

Contents of this file

1. Table S1
2. Figure S1
3. Figure S2

Introduction

This supporting information provides a table of the variables used to create a range of plausible sea-level rise scenarios, a figure depicting a simple diagnostic test that evaluates the reliability of our projections out to 2200, and a figure depicting historical observations from the Sewell's Point tide gauge.

Table S1: Comparison of key variables and information used to create a range of plausible scenarios with the National Research Council (1987) global mean sea-level rise model for USACE (2011, 2013, 2014), Parris et al. (2012), Hall et al. (2016), and Sweet et al. (2017).

**Study:**	**USACE [2011; 2013; 2014]**	**Parris et al. [2012]**	**Hall et al. [2016]**	**Sweet et al. [2017]**
Reference year	2000	2000	2000	2000
Eustatic SLC rate (mm/yr)	1.7	1.7	1.7	3
Local relative SLC rate (mm/yr)	4.44	4.44	4.44	
Rate of VLM	2.61	2.61	2.61	2.47
Constant b (m/yr^2)	2.71^{-5}<br>1.13^{-4}	2.71^{-5}<br>8.71^{-5}<br>1.56^{-4}	2.71^{-5}<br>7.00^{-5}<br>1.13^{-4}<br>1.56^{-4}	2.00^{-5}<br>7.00^{-5}<br>1.20^{-4}<br>1.70^{-4}<br>2.20^{-4}
Eustatic SLC in 2100 (m)	0.17 - 1.48	0.17 - 1.98	0.17 - 1.98	0.3 - 2.5
Local relative SLC in 2100 (m)	0.44 - 1.76	0.44 - 2.25	0.44 - 2.25	0.61 - 3.53

Note. SLC: sea-level change; VLM: vertical land movement

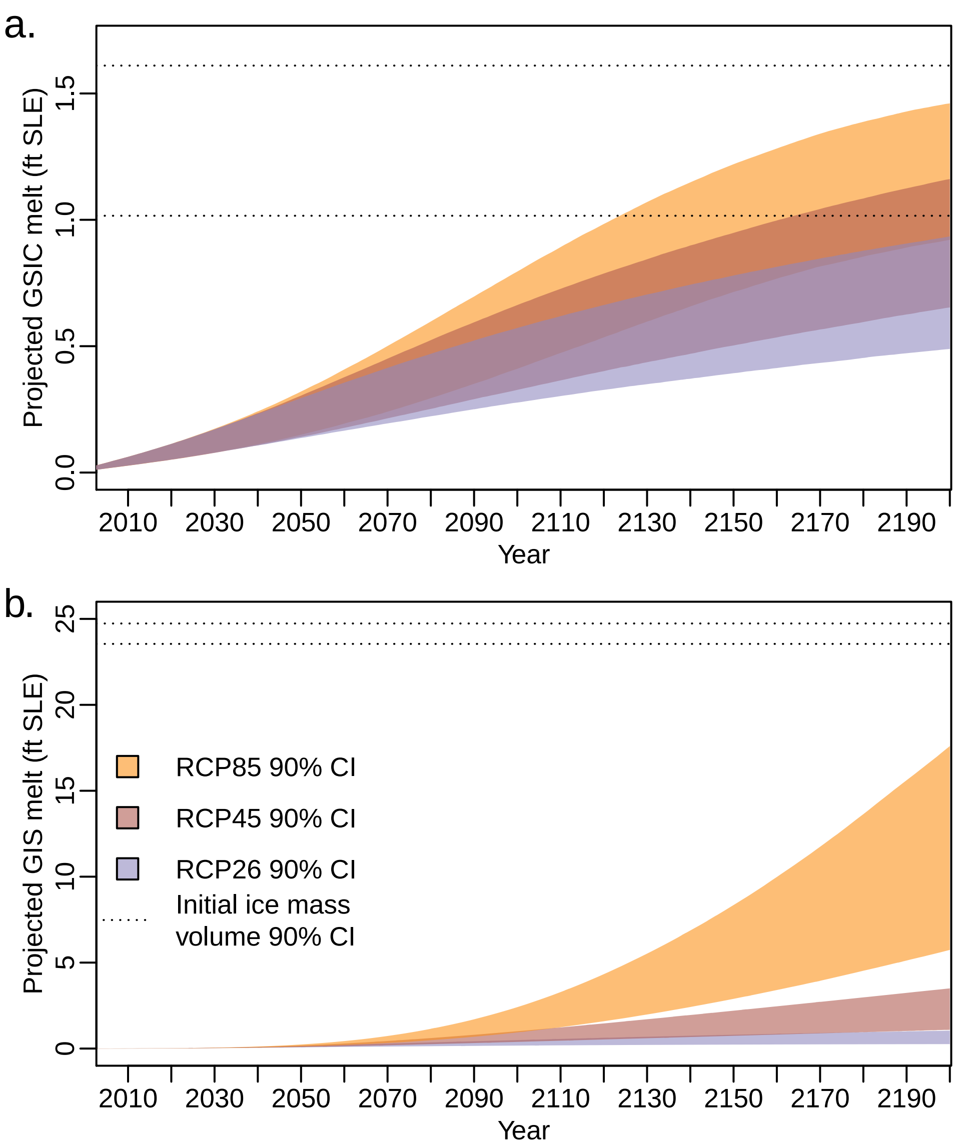

Figure S1: 90% credible intervals of ice mass projections (glaciers and small ice caps in panel a and Greenland ice sheet in panel b) in sea-level equivalence in comparison to the 90% credible interval of initial ice mass from *Wong et al.* [2017].

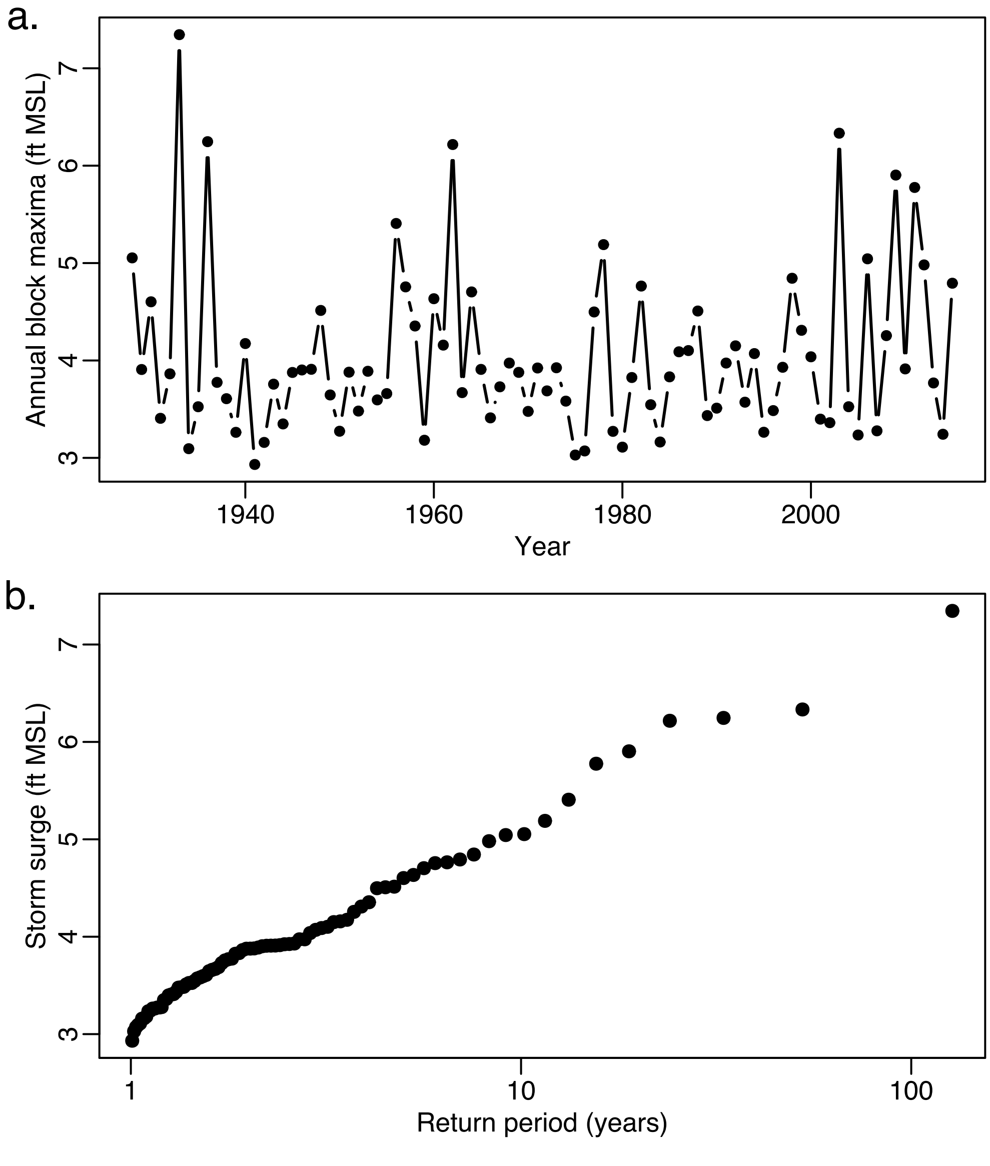

Figure S2: Maximum recorded sea-level anomaly in a year, the annual block maxima, for the Sewell's Point tide gauge (panel a) and the associated return periods for these storm surges (panel b).
